# The Role of Pre-Operative and Post-Operative Glucose Control in Surgical-Site Infections and Mortality

**DOI:** 10.1371/journal.pone.0045616

**Published:** 2012-09-19

**Authors:** Christie Y. Jeon, E. Yoko Furuya, Mitchell F. Berman, Elaine L. Larson

**Affiliations:** 1 School of Nursing, Columbia University School of Nursing, New York, New York, United States of America; 2 Center for Cancer Prevention and Control Research, University of California Los Angeles, Los Angeles, California, United States of America; 3 Division of Infectious Diseases, Columbia University Medical Center, New York, New York, United States of America; 4 Department of Anesthesiology, Columbia University Medical Center, New York, New York, United States of America; University of Texas Health Science Center at San Antonio, United States of America

## Abstract

**Background and Objective:**

The impact of glucose control on surgical-site infection (SSI) and death remains unclear. We examined how pre- and post-operative glucose levels and their variability are associated with the risk of SSI or in-hospital death.

**Methods:**

This retrospective cohort study employed data on 13,800 hospitalized patients who underwent a surgical procedure at a large referral hospital in New York between 2006 and 2008. Over 20 different sources of electronic data were used to analyze how thirty-day risk of SSI and in-hospital death varies by glucose levels and variability. Maximum pre- and post-operative glucose levels were determined for 72 hours before and after the operation and glucose variability was defined as the coefficient of variation of the glucose measurements. We employed logistic regression to model the risk of SSI or death against glucose variables and the following potential confounders: age, sex, body mass index, duration of operation, diabetes status, procedure classification, physical status, emergency status, and blood transfusion.

**Results:**

While association of pre- and post-operative hyperglycemia with SSI were apparent in the crude analysis, multivariate results showed that SSI risk did not vary significantly with glucose levels. On the other hand, in-hospital deaths were associated with pre-operative hypoglycemia (OR = 5.09, 95% CI (1.80, 14.4)) and glucose variability (OR = 1.14, 95% CI (1.03, 1.27) for 10% increase in coefficient of variation).

**Conclusion:**

In-hospital deaths occurred more often among those with pre-operative hypoglycemia and higher glucose variability. These findings warrant further investigation to determine whether stabilization of glucose and prevention of hypoglycemia could reduce post-operative deaths.

## Introduction

In 2002, an estimated 1.7 million healthcare-associated infections occurred in the U.S., 22% of which were surgical-site infections (SSI) that led to 8,205 deaths [1]. SSI is currently the most common healthcare-associated infection in developing countries where more than 30% of the operations result in SSI [2], and where prevalence of diabetes is also rapidly increasing [3]. Studies have shown that diabetes increases the risk of SSI, calling for careful monitoring of glucose levels in patients who undergo surgery [4], [5], a recommendation supported by the observation that peri- and post-operative hyperglycemia is associated with SSI [4], [5], [6].

Biological evidence demonstrates that diabetes can increase susceptibility to SSIs by compromising the immune system. For example, neutrophils from people with diabetes show reduced chemotaxis and oxidative killing potential compared to those from non-diabetic controls [7]. Leukocyte bactericidal activity is diminished in those with poor glucose control [8].

Given the adverse impact of diabetes on surgical outcomes, some have proposed tight glucose control using intensive insulin therapy (80 mg/dL−110 mg/dL) for all patients (diabetic or non-diabetic) scheduled to undergo a surgical procedure; however, this has shown no clear benefit in preventing SSIs or deaths in multiple randomized controlled trials [9], [10]. Existing guidelines on glucose control in inpatients and critically ill patients disagree on recommended target glucose levels, with minimum thresholds ranging from 80 mg/dL to 140 mg/dL and maximum thresholds ranging from 110 mg/dL to 200 mg/L [11], [12], [13], [14], [15]. A number of observational studies on the impact of pre-, peri-, or post-operative glucose control have found that post-operative hyperglycemia is associated with increased risk of SSIs [4], [6], [16], [17], [18] while the evidence for pre- or peri-operative glucose control has been equivocal [5], [9], [19]. The studies on post-operative glucose control have been limited in that the temporal order of the glucose measurement and SSI were not clear, confounders were not always considered, the studies varied on recommended targets for optimal glucose control for the preventions of SSIs, and death, a competing outcome to SSI, was not simultaneously considered. Furthermore, most studies have not investigated the role of variations in glucose levels, which has been found to predict SSIs, along with absolute glucose levels [20]. In light of the current state of controversy on the impact, target and timing of glucose control for prevention of SSI and death, we examined how the level of pre- and post-operative glucose and their variations are associated with the risk of SSI and death in inpatients.

## Methods

### Study Setting and Data

We undertook a retrospective cohort study employing de-identified data from a large adult academic tertiary healthcare facility in upper Manhattan. The data included 20,573 patients who were discharged between Jan 1, 2006 to Dec 31, 2008, and underwent an operative procedure categorized by the Center for Disease Control and Prevention’s National Healthcare Safety Network (NHSN) [21]. Data were extracted from: (1) the Clinical Data Warehouse that integrates information from over 20 clinical electronic sources shared by the three healthcare facilities; (2) the admission, discharge, transfer (ADT) system; (3) perioperative data system; and (4) anesthesiology data system. For the purpose of the present study, we included laboratory data on blood glucose and hemoglobin A1c (HbA1c) measurements and microbiologic results for infections. From the ADT system, we included demographic data on age and sex, ICD-9-CM diagnoses and procedure codes on diabetes status and NHSN procedure classifications. Furthermore, we included data on time and duration of operation from the perioperative records and data on blood transfusion, body mass index in units of kg/m^2^ (BMI), American Society of Anesthesiologists (ASA) physical status classification, and emergency status from the anesthesiology records.

### Diabetes and Glucose Control

Patients were considered to have diabetes if they ever had a discharge diagnosis of diabetes mellitus (ICD-9 250.00–250.99) during the study period. We determined the degree of glucose control based on blood glucose values available as part of a panel of metabolic indicators from blood drawn from the patients throughout their stay at the hospital. We considered pre-operative and post-operative glucose control, as well as long-term glucose control as measured by HbA1c. Pre-operative glucose control was determined by the maximum glucose value in the 72 hour period prior to operation, and post-operative glucose control was determined by the maximum value within the 72 hour period after the completion of operation. We classified the blood glucose levels into six categories: <80, 80–109, 110–139, 140–179, and ≥180 mg/dL based on recommended glucose targets from published guidelines for glucose control in inpatients or patients undergoing surgery [11], [12], [13], [14], [15]. Glucose categorized as 80–109 mg/dL based on maximum values were reassigned to <80 mg/dL if the patient ever had glucose levels of <80 mg/dL in the respective period of glucose assessment. Glucose variability was determined by computing the coefficient of variation of glucose values available in the pre- and post-operative period. We also summarized the difference in the maximum and minimum glucose values as an alternative measure of glucose variability. A minimum of 2 values were required to compute the coefficient of variation. Additionally, we defined long-term glucose control by the maximum HbA1c value available from 60 days before to 2 days after admission.

### Surgical Site Infections and In-hospital Death

SSI were identified as previously described [22], using an electronic definition modified from NHSN criteria. SSI were defined as occurring in patients who underwent a NHSN operative procedure and subsequently had a positive wound culture within 30 days of NHSN procedure as evident from discharge and in-hospital laboratory records. Microbiology cultures with key words ‘wound’, ‘abscess’, ‘surgical’, ‘drainage’, ‘body fluid’ or ‘surgical specimen’ in the specimen source indicated potential SSIs. Since colony forming units (CFU) are not routinely reported for cultures taken from these sites, a growth of any organism was considered a positive culture. In cultures which grew common skin contaminants (e.g. coagulase negative staphylococci, diphtheroids, etc.), those with at least two positive cultures on separate occasions were considered to be potential infections. Time of infection was determined by the day of culture collection. We defined in-hospital death as death due to any cause during hospital stay. Those who were readmitted with a SSI or died in a subsequent hospitalization were also counted as cases if the event occurred within 30 days of the operation. Patients who died or had a positive wound culture within three days of the operation were excluded from the analysis.

### Statistical Analysis

We compared the pre- and post-operative glucose level and glucose variability by SSI and death status by Wilcoxon rank sum test. We estimated the relative odds of SSI and death by logistic regression. We performed univariate analyses to determine the magnitude of unadjusted associations between SSI or death and the following: pre-operative and post-operative glucose levels, HbA1c, age, sex, BMI, operation duration, diabetes status, surgical type, ASA physical status classification, emergency status, and number of units of blood transfusion. We then conducted multivariate analyses to evaluate the associations between pre- or post-operative glucose or HbA1c levels and SSI or death, adjusting for the variables above that were significant at p<0.10 in univariate analyses. Where significant associations were detected for pre-operative or post-operative glucose levels, we further included the coefficient of variation of the glucose values in the models to determine how glucose variability is associated with SSI or in-hospital death independent of absolute glucose level. Significance of associations was interpreted at p-value of <0.05. All statistical analyses were performed with SAS Version 9.2 (SAS Institute, Cary, NC).

This study was approved by the Institutional Review Board of Columbia University Medical Center.

## Results

Of 20,573 patients who underwent a NHSN operative procedure according to discharge records, we matched 16,288 patients to data available in perioperative and anesthesiology databases. We ultimately analyzed data on 13,800 patients for whom pre-operative (n = 5,618) or post-operative (n = 13,166) glucose levels were available, 21.2% (n = 2,927) of whom had diabetes. Analyses examining long-term glucose control were limited to 2,882 individuals for whom we had HbA1c data. The median age of the patients was 61, and 47% were female. Abdominal and cardiac procedures comprised half of the surgical types. A majority of patients were classified as ASA physical status of 2 or 3, underwent an elective surgery, and did not require blood transfusion. Post-operative glucose levels were higher than pre-operative glucose levels, with over 65% of patients experiencing glucose levels of > = 140 mg/dL after operation. We observed 260 culture positive SSIs, and 232 in-hospital deaths, of which 16 occurred on the day of the infection or later. Patients with SSIs and those who died had higher maximum pre-operative and post-operative glucose levels compared to others (SSI: p = 0.04 for pre-operative and <0.0001 for post-operative; death: p<0.0001 for pre-operative, p<0.0001 for post-operative). Glucose variability as measured by coefficient of variation was also higher in those with SSI and those who died (SSI: p = 0.13 pre-operative and <0.0001 for post-operative; death: p<0.0001 for pre-operative and p<0.0001 for post-operative) (Table 1).

**Table 1:** Characteristics of patients who underwent surgical procedures by status of infection and in-hospital death.

Variables	Categories	All patients		Surgical site infections		Death	
		N	Median (IQR)	N	Median (IQR)	N	Median (IQR)
**Continuous variables**							
Age (years)		13800	61 (48, 72)	260	63 (51, 74)	232	70 (57, 80)
BMI (kg/m^2^)		13800	26.6 (23.4, 30.3)	260	26.7 (23.7, 31.4)	232	25.8 (22.8, 29.9)
Operation duration (min)		13772	193 (115, 280)	258	251 (127, 367)	231	202 (105, 332)
Pre-operative glucose (mg/dL)							
	Maximum glucose	5618	117 (98, 150)	182	123 (99, 176)	182	138 (110, 184)
	Coefficient of variation (%)	3133	13.2 (6.7, 23.0)	125	15.8 (7.2, 25.8)	140	16.7 (10.5, 29.5)
	Difference in max and min	3133	27 (12, 55)	125	37 (15, 67)	140	41 (20, 85)
Post-operative glucose (mg/dL)							
	Maximum glucose	13166	158 (130, 193)	255	180 (144, 221)	231	185 (155, 242)
	Coefficient of variation (%)	11026	20.8 (13.6, 29.5)	242	24.4 (16.6, 33.5)	229	27.2 (19.5, 37.3)
	Difference in max and min	11026	61 (35, 99)		82 (51, 128)		97 (62, 156)
Hemoglobin A1C (%)		2882	6.1 (5.6, 6.9)	69	6.4 (5.7, 7.9)	71	6.2 (5.7, 7.8)
**Categorical** **Variables**		**N**	**% among total**	**N**	**% SSI among those with covariate**	**N**	**% death among those with covariate**
Sex							
	Female	6546	47.4%	103	1.6%	114	1.7%
	Male	7254	52.6%	157	2.2%	118	1.6%
Diabetes mellitus							
	Complicated	1554	11.3%	66	4.2%	48	3.1%
	Un-complicated	1781	12.9%	43	2.4%	46	2.6%
	No	10465	75.8%	151	1.4%	138	1.3%
Malignancy							
	Yes	3483	25%	48	1.4%	60	1.7%
	No	10317	75%	212	2.1%	172	1.7%
Surgery Types*							
	Abdominal	3612	26.2%	113	3.1%	66	1.8%
	Cardiac	3309	24.0%	69	2.1%	81	2.4%
	Neurological	2249	16.3%	23	1.0%	47	2.1%
	Genitourinary	1837	13.3%	13	0.7%	3	0.2%
	Orthopedic	1754	12.7%	53	3.0%	23	1.3%
	Thoracic	1118	8.1%	17	1.5%	43	3.8%
	Gynecological	554	4.0%	6	1.1%	4	0.7%
	Vascular	453	3.3%	21	4.6%	8	1.8%
	Herniorrhaphy	364	2.6%	11	3.0%	9	2.5%
	Ear, Nose, Throat	150	1.1%	0	0.0%	1	0.7%
	Breast	96	0.7%	0	0.0%	1	1.0%
ASA physical status							
	0–1	762	5.5%	5	0.7%	1	0.1%
	2	5709	41.4%	54	0.9%	13	0.2%
	3	6109	44.3%	156	2.6%	111	1.8%
	4+	1221	8.8%	45	3.7%	91	7.5%
Emergency							
	Yes	2121	15.4%	79	3.7%	93	4.4%
	No	11679	84.6%	181	1.5%	139	1.2%
Blood transfusion units							
	0	12166	88.2%	192	1.6%	160	1.3%
	1	908	6.6%	27	3.0%	27	3.0%
	2+	726	5.3%	41	5.6%	45	6.2%
Pre-operative glucose (mg/dL)							
	<80 mg/dL	207	3.7%	7	3.4%	9	4.3%
	80–109 mg/dL	2121	37.8%	63	3.0%	36	1.7%
	110–139 mg/dL	1534	27.3%	37	2.4%	50	3.3%
	140–180 mg/dL	911	16.2%	33	3.6%	37	4.1%
	> = 180 mg/dL	845	15.0%	42	5.0%	50	5.9%
Post-operative glucose (mg/dL)							
	<80 mg/dL	110	0.8%	2	1.8%	0	0.0%
	80–109 mg/dL	1340	10.2%	15	1.1%	8	0.6%
	110–139 mg/dL	2976	22.6%	42	1.4%	30	1.0%
	140–180 mg/dL	4429	33.6%	68	1.5%	70	1.6%
	> = 180 mg/dL	4311	32.7%	128	3.0%	123	2.9%

* A patient may belong to more than one category.

IQR = interquartile range, SSI = surgical-site infection.

In univariate analyses, pre- and post-operative glucose levels of > = 180 mg/dL were associated with SSI. Pre-operative glucose level of > = 110 mg/dL, pre-operative hypoglycemia, and post-operative glucose level of > = 140 mg/dL were associated with in-hospital death. Each 10% increase in the coefficient of variation of pre-operative and post-operative glucose levels was also associated with 11–23% increased risk of SSI and in-hospital deaths. In addition, each 1% increase in HbA1c levels was associated with SSI, but not with in-hospital death. (Table 2).

**Table 2:** Relationship between demographic, clinical and operative factors and surgical site infections and death.

Variables	Categories	Univariate for SSI			Univariate for death		
		OR	95% CI	p-value	OR	95% CI	p-value
Age (increase in 10 years)		1.08	1.00, 1.17	0.04	1.43	1.31, 1.57	<0.0001
Sex							
	Female	0.72	0.56, 0.93	0.01	1.07	0.83, 1.39	0.60
	Male	Ref			Ref		
BMI (increase 1 kg/m^2^)		1.013	0.995, 1.031	0.15	0.99	0.97, 1.01	0.48
Diabetes mellitus							
	Complicated	3.03	2.26, 4.07	<0.0001	2.39	1.71, 3.33	<0.0001
	Uncomplicated	1.69	1.20, 2.38		1.98	1.42, 2.78	<0.0001
	No	Ref			Ref		
Malignancy							
	Yes	0.79	0.59, 1.07	0.13	1.03	0.77, 1.39	0.82
	No	Ref			Ref		
Surgical type (> = 3% frequency)*							
	Abdominal	2.21	1.72, 2.83	<0.0001	1.12	0.84, 1.50	0.43
	Cardiac	1.15	0.87, 1.52	0.33	1.72	1.31, 2.26	0.0001
	Neurological	0.49	0.32, 0.76	0.001	1.31	0.95, 1.81	0.10
	Genitourinary	0.34	0.19, 0.59	0.0001	0.08	0.03, 0.26	<0.0001
	Orthopedic	1.78	1.31, 2.42	0.0002	0.75	0.49, 1.16	0.20
	Thoracic	0.79	0.48, 1.30	0.35	2.64	1.89, 3.70	<0.0001
	Gynecological	0.56	0.25, 1.26	0.16	0.42	0.15, 1.12	0.08
	Vascular	2.67	1.69, 4.21	<0.0001	1.05	0.52, 2.15	0.89
	Herniorrhaphy	1.65	0.89, 3.05	0.11	1.50	0.77, 2.95	0.24
	Ear, Nose, Throat	0			0.39	0.05, 2.80	0.35
	Breast	0			0.61	0.09, 4.42	0.63
ASA physical status							
	0–1	0.69	0.28, 1.74	0.43	0.54	0.07, 4.07	0.55
	2	Ref			Ref		
	3	2.75	2.01, 3.75	<0.0001	8.15	4.68, 14.2	<0.0001
	4+	4.01	2.69, 5.98	<0.0001	35.1	20.0, 61.7	<0.0001
Emergency							
	Yes	2.46	1.88, 3.21	<0.0001	3.81	2.92, 4.97	<0.0001
	No	Ref			Ref		
Operation time (increase in 10 min)		1.022	1.015, 1.029	<0.0001	1.012	1.004, 1.021	0.004
Blood transfusion units							
	0	Ref			Ref		
	1	1.91	1.27, 2.88	0.002	2.30	1.52, 3.48	<0.0001
	2+	3.73	2.64, 5.28	<0.0001	4.96	3.53, 6.96	<0.0001
Pre-operative glucose (mg/dL)							
	Maximum glucose (increase10 mg/dL)	1.02	1.01, 1.03	0.009	1.03	1.02, 1.04	<0.0001
	Coefficient of variation(increase in 10%)	1.11	1.02, 1.21	0.02	1.19	1.11, 1.28	<0.0001
	<80 mg/dL	1.14	0.52, 2.53	0.74	2.63	1.25, 5.54	0.01
	80–109 mg/dL	Ref			Ref		
	110–139 mg/dL	0.81	0.54, 1.22	0.31	1.95	1.27, 3.01	0.003
	140–180 mg/dL	1.23	0.80, 1.89	0.35	2.45	1.54, 3.91	0.0002
	> = 180 mg/dL	1.71	1.15, 2.55	0.009	3.64	2.36, 5.63	<0.0001
Post-operative glucose (mg/dL)							
	Maximum glucose (increase10 mg/dL)	1.02	1.01, 1.03	<0.0001	1.03	1.02, 1.04	<0.0001
	Coefficient of variation(increase in 10%)	1.15	1.07, 1.21	<0.0001	1.23	1.16, 1.31	<0.0001
	<80 mg/dL	1.53	0.35, 6.75	0.57	0		
	80–109 mg/dL	Ref			Ref		
	110–139 mg/dL	1.19	0.66, 2.11	0.57	1.70	0.78, 3.71	0.19
	140–180 mg/dL	1.27	0.73, 2.20	0.39	2.67	1.28, 5.57	0.009
	> = 180 mg/dL	2.53	1.50, 4.27	0.0005	4.89	2.39, 10.0	<0.0001
Hemoglobin A1c (%)		1.17	1.04, 1.31	0.01	1.08	0.94, 1.23	0.28

* a patient may belong to more than one NHSN surgery category.

SSI = surgical-site infection.

Adjusting for age, sex, diabetes, surgical type, ASA physical status, emergency, operation time, and blood transfusion, the association between pre- and post-operative glucose levels and SSI was attenuated to non-significant relationship. In multivariate analysis, adjusting for age, diabetes, surgical type, ASA physical status, emergency, duration of operation, and blood transfusion, pre-operative glucose levels of <80 mg/dL (OR = 2.55, 95% CI 1.17, 5.59), 110–139 mg/dL (OR = 1.65, 95% CI 1.05, 2.58, and > = 180 mg/dL (OR = 1.91, 95% CI 1.17, 3.10) remained significantly associated with in-hospital deaths. However, when we included coefficient of variation of pre-operative glucose levels in the model, glucose levels of 110 mg/dL or above were not significantly associated with death, while glucose level of <80 mg/dL was strongly associated with in-hospital death (OR = 5.09, 95% CI 1.80, 14.4). Additionally, each 10% increase in coefficient of variation of pre-operative glucose levels were associated with in-hospital mortality (OR = 1.14, 95% CI 1.03, 1.27). In the multivariate analyses, the association between post-operative glucose levels and in-hospital death became non-significant, although a dose-response effect was apparent (p = 0.04 for trend). The dose response trend disappeared when we adjusted for the coefficient of variation of post-operative glucose levels. Post-operative glucose variability was associated with death (OR = 1.17, 95% CI 1.08, 1.28 for each 10% increase in coefficient of variation). In addition, HbA1c levels were not associated with either SSI or death in the multivariate models. (Table 3).

**Table 3:** Multivariate association between demographic, clinical and operative factors and surgical site infections and death.

Variables	Categories	Multivariate for SSI			Multivariate for death		
		OR	95% CI	p-value	OR	95% CI	p-value
Pre-operative glucose (mg/dL)*							
	<80 mg/dL	0.99	0.44, 2.23	0.97	2.55	1.17, 5.59	0.02
	80–109 mg/dL	Ref			Ref		
	110–139 mg/dL	0.76	0.50, 1.16	0.20	1.65	1.05, 2.58	0.03
	140–180 mg/dL	1.06	0.67, 1.67	0.81	1.57	0.96, 2.57	0.07
	> = 180 mg/dL	1.21	0.77, 1.91	0.41	1.91	1.17, 3.10	0.009
Post-operative glucose (mg/dL)**							
	<80 mg/dL	1.41	0.312, 6.31	0.65	0		
	80–109 mg/dL	Ref			Ref		
	110–139 mg/dL	1.28	0.71, 2.30	0.42	1.40	0.63, 3.10	0.41
	140–180 mg/dL	1.30	0.73, 2.31	0.37	1.61	0.75, 3.43	0.22
	> = 180 mg/dL	1.72	0.97, 3.06	0.07	1.89	0.88, 4.06	0.10
Hemoglobin A1c (1% increase)***		0.97	0.84, 1.13	0.70	0.93	0.77, 1.12	0.45

SSI = surgical-site infection.

* ORs for pre-operative glucose levels estimated from model restricted to those with pre-operative glucose levels available.

** ORs for post-operative glucose levels estimated from model restricted to those with post-operative glucose levels available.

*** ORs for hemoglobin A1c levels estimated from model restricted to those with hemoglobin A1c levels available.

Covariates positively associated with SSI in multivariate analysis included diabetes with complications (OR = 1.55, 95% CI 1.11, 2.17), abdominal surgery (OR = 3.01, 95% 2.17, 4.18), orthopedic surgery (OR = 4.86, 95% CI 3.23, 7.31), vascular surgery (OR = 3.01, 95% CI 1.83, 4.96), ASA physical status of 3 and 4 (OR = 2.32, 95% CI 1.64, 3.30), OR = 2.87, 95% CI 1.78, 4.65, respectively), emergent procedure (OR = 1.41, 95% CI 1.02, 1.94), operation time (OR = 1.012, 95% CI 1.005, 1.022 for 10 min increase). Those who underwent neurological procedures were less likely to experience SSI, compared to those who underwent other types of surgery (OR = 0.47, 0.29, 0.77).

Covariates positively associated with in-hospital death in multivariate analysis included age (OR = 1.28, 95% CI 1.16, 1.41 for 10 year increase), thoracic surgery (OR = 2.30, 95% CI 1.60, 3.30), ASA physical status of 3 and 4(OR = 5.48 95% CI 3.08, 9.75, OR = 17.01, 95% CI 9.13, 31.7, respectively), emergent procedure (OR = 2.41, 95% CI 1.75, 3.33), and two or more units of blood transfusion (OR = 1.86, 95% CI 1.23, 2.81). Those who underwent genitourinary procedure were less likely to experience in-hospital death than those who underwent other procedures (OR = 0.14, 95% CI 0.05, 0.46).

## Discussion

In this study of 13,800 hospitalized patients who underwent surgery, we found that in-hospital deaths occurred more often in individuals with hypoglycemia and higher glucose variability in the pre-operative period. On the other hand, we found no association between low or high levels of glucose and SSI.

Consistent with our findings, studies by Meyfroidt et al. and Dungan et al. also found that variation in glucose was associated with mortality, independent of blood glucose level. [20], [23] Glycemic variability may impact one’s risk of death by way of elevated production of free radicals, which has been implicated in vascular damage [24]. Severe hypoglycemia could have neurological effects with fatal consequences, including cardiac arrhythmia [25]. Low glucose levels or sudden variations in glucose may also indicate the severity of illness associated with mortality risk. Critical illness leads to both enhanced glucose production and utilization [26] that may cause erratic changes in glucose levels. Also, hypoglycemia is correlated with higher levels of albumin, which signal renal and hepatic dysfunction [27].

Although we observed an apparent association between pre- and post-operative hyperglycemia and SSI in the crude analysis, as had been found in other studies [16], [17], [18], our multivariate results did not substantiate these associations. We controlled for multiple factors, including age, sex, diabetes, surgical type, physical status, emergency procedure, duration of operation and blood transfusion units that collectively confounded the association between glucose levels and SSIs. It is possible that elevations in post-operative glucose levels among those who developed SSI may reflect the natural glycemic dysregulation that occurs with surgery, the extent to which could be correlated with specific procedures that increase the risk of infections [28].

The strengths of our study include a large sample size of inpatients which allowed us to investigate risk factors of SSI and in-hospital deaths, which occur infrequently, and the inclusion of a comprehensive group of patients who underwent a variety of surgical procedures. Our findings are therefore generalizable to a broader population undergoing various types of surgery. Access to expansive sources of electronic data made it possible to measure and control for multiple confounders. In addition, we assessed glucose levels prior to the period in which SSI and death were ascertained, to establish temporal order of exposure and outcome of interest. This is important considering that patients experiencing critical infections could have altered glucose homeostasis [29]. Lastly, we examined both SSI and death simultaneously since death could be a competing outcome for SSI. Because pre-operative glucose levels were associated with mortality, it is possible that associations between pre-operative glucose levels and SSI may be influenced by survivors bias, a factor which has not been explored in previous studies.

Despite its strengths, the study also had a number of limitations. First, a large number of patients lacked measurements of pre-operative glucose levels and their variation, because operations occurred often on the day of the admission. This may have limited our power to analyze the association between death and pre-operative glucose levels and their variation simultaneously. Our analyses for long-term glucose control were also limited in power and generalizability, because only 21% of our study population had data on HbA1c. Further studies examining the impact of long-term glucose control and its variation on surgical-site-infection would be valuable. Also, we did not have information on peri-operative glucose levels. Further, we only detected SSIs and deaths that occurred during hospitalization; thus, cases that occurred in outpatient settings or outside the healthcare system could not be assessed. While it is difficult to predict the direction of bias under such selective case ascertainment, it is possible that our results for hypoglycemia and glucose variation may have been biased upwards if patients with stable normoglycemic glucose levels were less likely to have longer hospital stays and to return for readmission. Moreover, we lacked data on specific causes of death, thus it remains unclear whether stabilizing glucose level would have prevented the specific conditions that lead to in-hospital deaths.

In conclusion, we found that pre-operative hypoglyemia and glucose variation are associated in-hospital morality. If the association reflects a causative relationship, glucose stabilization and prevention of hypoglycemia could be important modifiable intervention for reduction in in-hospital mortality. Further studies with large sample sizes, optimal control for confounding, and establishment of temporal order of glucose measurement and SSIs are needed to determine the association between glucose control and SSI.
